# *In vitro* and *In vivo* Antioxidant, Anti-hyperlipidemic Properties and Chemical Characterization of *Centella asiatica* (L.) Extract

**DOI:** 10.3389/fphar.2016.00400

**Published:** 2016-10-28

**Authors:** Sima Kumari, Meetali Deori, R. Elancheran, Jibon Kotoky, Rajlakshmi Devi

**Affiliations:** ^1^Biochemistry Laboratory, Life Sciences Division, Institute of Advanced Study in Science and TechnologyGuwahati, India; ^2^Drug Discovery Laboratory, Life Sciences Division, Institute of Advanced Study in Science and TechnologyGuwahati, India

**Keywords:** *Centella asiatica*, antioxidant, anti-hyperlipidemic, medicinal plants, UHPLC–MS/MS

## Abstract

The study aimed to identify the phenolic compounds present in *Centella asiatica* (L.) (*C. asiatica*) extract and evaluate the respective antioxidant potential as well as its cholesterol-lowering effects in the experimental animal model. Herein, the antioxidant potential of extracts was assessed by its free radical scavenging activity such as 2, 2-diphenyl -1- picrylhydrazyl as well as reducing capability. The anti-hyperlipidemic effects of *C. asiatica* extract (CAE) were evaluated in high cholesterol-fed (HCF) rats for 4 weeks, where different concentrations of extracts (0.25, 0.5, and 1 g/kg/day) were orally administrated daily. Lipid and antioxidant profiles, including total cholesterol (TC), triglyceride (TG), low density lipoprotein cholesterol (LDL-C), high density lipoprotein cholesterol (HDL-C) and superoxide dismutase (SOD), together with the indices of hepatic functions were also examined. *C. asiatica* revealed excellent free radical scavenging activity as revealed by 2-2- diphenyl-1-picryl-hydrazyl (DPPH) assay, with the IC_50_ values (9.62 ± 0.88 μg/mL). Furthermore, *C. asiatica* extracts and fenofibrate remarkably lowered the level of TC, TG, LDL-C, and showed elevated levels of HDL-C, SOD. The histopathological observations further demonstrated clear differentiation and structural changes in liver of HCF and CAE treated group. Furthermore, gulonic acid, ferulic acid, kaempferol, chlorogenic acid, and asiatic acid were identified to be the major components which might be responsible for the antioxidant activity of the *C. asiatica* extract as evidenced from an ultra-high performance liquid chromatography–mass spectrometer. Taken together, these results signifies the excellent antioxidant and anti-hyperlipidemic properties of *C. asiatica* leaf extracts, which might be useful for the treatment of oxidative-stress related diseases such as hyperlipidemia.

## Introduction

Oxidative stress (OS) is essentially an imbalance between the production of free radicals, such as superoxide ($O_{2}^{–}$), hydroxyl (OH), and peroxyl (ROO) radicals and the ability of the body to counteract or detoxify their harmful effects through neutralization by antioxidants. OS leads to many chronic and degenerative diseases such as cancer, atherosclerosis, Parkinson’s, Alzheimer’s, diabetes, neurodegenerative disorders, and aging (Yu, 1994; Raghuveer and Tandon, 2009). Overproduction of these radicals can lead to the above mentioned disorders with as a consequence of “OS/oxidative damage.” In earlier reports, various synthetic antioxidant compounds such as butylated hydroxyanisole (BHA), butylated hydroxytoluene (BHT), tertiary butylhydroquinone (TBHQ), and propyl gallate (PG) have shown effectiveness in the human body against oxidative damage caused by free radicals (Issa et al., 2006; Kaliora et al., 2006). However, the utilization of synthetic antioxidants is restricted now a day due to their toxicity and undesirable effects on human health (Sasaki et al., 2002; Bajpai et al., 2014).

Hyperlipidemia is represented by elevated levels of plasma lipid, serum cholesterol, and triglyceride (TG) and remains one of the major risk factors for coronary heart diseases (CHD), especially, the increased plasma low-density lipoprotein level can accelerate atherosclerosis (Arsenault et al., 2009; Harchaoui et al., 2009; de Lemos et al., 2010; Berry et al., 2012; Ma et al., 2012). The excessive intake of exogenous TG and cholesterol is associated with an elevated risk of atherosclerotic disease, which is regarded as a lipid storage disease with arterial deposition of cholesterol, endothelial injury and platelet aggregation (Liao et al., 2000). Several research groups have demonstrated that with the improvement of serum lipid profile, such as total cholesterol (TC), TG, and (TC-HDL-C)/HDL-c decreases liver lipid accumulation and lipid absorption. Therefore, the increasing serum/liver antioxidant capacity represents the most effective way for combating the occurrence of cardiovascular or liver disorders (Yang D.J. et al., 2010; Yang S.F. et al., 2010; Chang et al., 2011; Hsu et al., 2011; Liu et al., 2012; Lin et al., 2013).

Medicinal plants are rich sources of important metabolites, which are potential sources of antioxidant, antimicrobial, anti-inflammatory, and anticancer activities (Hernandez et al., 2000; Polya, 2003; Maruthanila et al., 2014). The utilization of herbal medicine in treating infectious diseases have been practiced for 1000s of years and will continue to provide mankind with new remedies (Cragg and Newman, 2007). Earlier reports have demonstrated a distinct correlation between the higher intake of plant foods and lower risk of mortality from diseases (Chidambaram et al., 2013). Recent interest has escalated in the finding antioxidant properties of natural origin plants due to their superior safety and consumer acceptability (Gorinstein et al., 2003). Additionally, approximately 60% of the commercially available anti-tumor and anti-infective agents are natural in origin (Shridhar et al., 2009). North Eastern (NE) region of India represents one of the mega biodiversity hotspots of the world and harbors a large number of medicinal plants. Around 80% of the population relies mainly on traditional medicine for their primary healthcare needs due to the high cost of western medicine (Rai and Lalramnghinglova, 2010; Lokho, 2012).

*Centella asiatica* (Family, Apiaceae) is commonly known as *Centella* and Gotu kola. It is native to India and other parts of Asia such as China, Sri Lanka, Nepal and Madagascar and has been utilized in folk medicine in different countries (Gohil et al., 2010). *C. asiatica* has been reported for the treatment of varicose veins, chronic venous insufficiency, psoriasis, minor wounds, analgesic, and anti-inflammatory effects (Pronprapa et al., 2010; Somboonwong et al., 2012). The primary constituents of *C. asiatica* are saponins (also called triterpenoids), which include asiaticosides, brahmoside, asiatic acid, and brahmic acid (madecassic acid) (Joshi and Chaturvedi, 2013). These triterpene saponins and their sapogenins are mainly responsible for the wound healing and vascular effects, which acts by inhibiting the production of collagen at the wound site (Seevaratna et al., 2012; Joshi and Chaturvedi, 2013). Other components isolated from *C. asiatica*, such as brahmoside and brahminoside, may be responsible for central nervous system (CNS) and uterorelaxant actions, but are yet to be confirmed by clinical studies (Orhan, 2012; Seevaratna et al., 2012; Joshi and Chaturvedi, 2013). In a recent study, researchers have utilized *C. asiatica* for wound healing activities of different extracts in partial-thickness burn wound models in rats (Somboonwong et al., 2012). In our previous report, we have identified and characterized phenolic compounds in *C. asiatica* by using UHPLC-MS/MS (Kumari et al., 2016). However, its anti-hyperlipidemic activities in rat fed with high-fat diet have not been documented yet from NE region. Therefore, we have designed this study in order to demonstrate the anti-hyperlipidemic activity of *C. asiatica* extracts in normal and high cholesterol-fed (HCF)-induced hyperlipidemic rat, which is the crucial factor in the treatment of oxidative-stress related diseases.

## Materials and Methods

### Sample Collection

*Centella asiatica* plant was collected from the Sixmile, Guwahati, and Kamrup district of Assam (situated in between 25° 43′–26° 53′ North latitude and 90° 39′–92° 11′ East latitude in the month of June to August 2015). The plant was authenticated and reconfirmed in the Department of Botany, Gauhati University, Assam. Herbarium *C. asiatica* (voucher specimens number: IASST/BCCS/HNO21/2013), was deposited in the Life Science Division (LSD) of the Institute of Advanced Study in Science and Technology (IASST), Guwahati, India for future references.

### Sample Preparation

Freshly collected plant (*C. asiatica*) was washed twice with distilled water, dried at room temperature, powdered and used for extraction. The dried plant powder (100 g) was dissolved in 1000 mL of distilled water for 72 h with occasional shaking. The extract was further filtered through Whatman No. 1 filter paper and the filtrate was concentrated using a bench top lyophilizer under reduced pressure. The extract was stored at 4°C until further use in the designated experiment.

### Chemicals and Reagents

Standards, such as DPPH, 2,2′-azinobis-3-ethyl benzothiazole-6-sulfonic acid (ABTS), trolox, ascorbic acid and standard hyperlipidemic drug, i.e., Fenofibrate and standard enzyme used for catalase, SOD and diagnostic kits for measuring TC, TG, high density lipoprotein- cholesterol (HDL-C), were purchased from Sigma-Aldrich. Methanol (Fischer Scientific) and ethyl acetate (Merck) were procured. Potassium ferricyanide, trichloroacetic acid (TCA), ferric chloride, sodium nitroprusside, naphthyl ethylenediamine dichloride, sulfanilamide, phosphoric acid, sodium hydroxide, sodium chloride, hydrochloric acid, ferrozine, and ferrous chloride were obtained from HiMedia laboratory, India.

### UHPLC-ESI Orbitrap MS/MS Analysis

The chromatographic separation was carried out using an ultra-high performance liquid chromatography (UHPLC) system coupled with an electron spray ionization (ESI) Orbitrap mass spectrometer. A series of 3100 UHPLC system (Dionex, Inc., Sunnyvale, CA, USA) is equipped with a binary pump, a degasser, an autosampler, a thermostated column compartment, and control module. The chromatographic separation was on a Hypersil Gold C18 column (1.9) operated at 25°C. Gradient chromatographic separation was performed for each extract of the samples by using the previous method (Kumari et al., 2016). The screening and identification of the phenolic compounds in the *C. asiatica* extract were determined by using mass spectrum and its unique fragmentation spectrum. The comparison of the observed MS/MS spectra with those found in the literature and mass bank database was the main tool for putative identification of the phenolic compounds.

### *In vitro* Measurement of Antioxidant Properties

#### 2, 2-Diphenyl -1- Picrylhydrazyl (DPPH) Radical Scavenging Activity Assay

The free radical scavenging activity of the samples was determined according to the method of Kumari et al. (2016). A freshly prepared solution of DPPH in methanol (6 × 10^-5^ M) was used for the UV measurements. The samples of different concentrations (4–64 μg/mL) were added to DPPH solution in 1:1 ratio followed by vortexing. Then, it was allowed to take place in the dark at room temperature. Ascorbic acid and trolox were utilized as a standard. The inhibition percentage of DPPH radical scavenging activity was calculated using the following equation.

$$Inhibition(\%)\;=\;[(A_{0}-A)/A_{0}]\;\times \;100$$

Where, *A*_0_ is the absorbance of DPPH in the absence of the sample and *A* is the absorbance of DPPH in the presence of the sample.

The IC_50_ values (the concentration required to scavenge 50% of the free radical) were estimated from a plot of % inhibition against the concentration of the sample solutions.

#### Total Reduction Capability

The total reduction capability of samples was determined according to the method of Kumari et al. (2016). 2.5 mL of 0.2 M phosphate buffer (pH 6.6) and 2.5 mL of 1% potassium ferric cyanide were added to 1 mL of samples in different concentrations (4–64 μg/mL), followed by gentle mixing. The mixture was incubated at 50°C in a water bath for 20 min. The reaction was stopped by adding 2.5 mL of 10% TCA and the mixture was centrifuged at 4000 rpm for 10 min. From the top layer, 2.5 mL was transferred into the tube containing 2.5 mL distilled water and 0.5 mL of 0.1% ferric chloride (FeCl_3_.6H_2_O), mixed thoroughly. After 5 min, the absorbance was measured at 700 nm against blank. Trolox and ascorbic acid were taken as a standard.

#### ABTS^+^ Radical Cation Decolorization Assay

The antioxidant activities of the extracts were determined by the improved ABTS^+^ radical cation scavenging ability with the slight modification (Re et al., 1999; Sarma et al., 2016). ABTS^+^ radical cation was produced by mixing 7 mM 2, 2′- azino-bis (3-ethylbenzothiazoline-6-sulfonic acid) diammonium salt (ABTS) and 2.45 mM potassium persulfate (K_2_S_2_O_8_), incubated at room temperature in the dark. To determine the ABTS radical scavenging activity, 3 mL of ABTS^+^ solution was mixed thoroughly with 0.2 mL of different concentration (4–64 μg/mL) of extracts. Ascorbic acid and trolox were taken as a standard. The reaction mixture was allowed to stand at room temperature for 6 min.

The percentage inhibition was calculated by the following formula:

%Inhibition = OD of control−OD of sample/OD of control × 100

#### Hydrogen peroxide (H_2_O_2_) Radical Scavenging Activity

Hydrogen peroxide (H_2_O_2_) radical scavenging activities of the extracts were determined according to the method of Sarma et al. (2016). Briefly, water extracts of *C. asiatica* (4–64 μg/mL) were added to 0.6 mL H_2_O_2_ (40 mM) with the prepared phosphate buffer (pH 7.4). The reaction mixtures were incubated at room temperature for 10 min. After the incubation, the reaction mixture read at 230 nm against the blank solution with phosphate buffer (pH 7.4). Ascorbic acid and trolox were taken as a standard. The inhibition percentage was calculated based on the formula:

$$Percentage(\%)\;of\;inhibition\;=\;(A_{1}-A_{2})/A_{1}\;\times \;100.$$

Where, *A*_1_- absorbance of the H_2_O_2_ and *A*_2_- absorbance of the reaction mixture with extract.

#### Nitric Oxide (NO) Radical Scavenging Assay

Nitric oxide (NO) radical scavenging assay was carried out by the method of Kumari et al. (2016). 0.6 mL of 10 mM sodium nitroprusside was mixed with 1 mL of water extract of *C. asiatica* in different concentration (4–64 μg/mL). The mixture was incubated at 25°C for 150 min, followed by mixing with 1.0 mL of pre-prepared Griess reagent (1% sulfanilamide, 0.1% naphthyl ethylenediamine dichloride, and 2% phosphoric acid). Ascorbic acid and trolox were taken as a standard. The absorbance was measured at 546 nm. The inhibition was calculated by the following equation:

$$\%inhibition\;of\;NO\;radical\;=\;[A_{0}-A_{1}]/A_{0}\;\times \;100.$$

Where, *A*_0_ is the absorbance before the reaction and *A*_1_ is the absorbance after the reaction has taken place with Griess reagent.

The decreasing absorbance indicates a high NO scavenging activity.

#### *In vitro* Lipid Peroxidation (LPO) Assay

##### Preparation of rat liver homogenate

Adult Wistar albino rats (150–200 g) were anesthetized with sodium pentobarbitone (35 mg kg^-1^) followed by excision of one lobe of the liver and washing with 0.9% NaCl solution. Tissue homogenate was prepared in a ratio of 1 g wet tissue to 10 times (w/v) 0.05 M ice-cold phosphate buffer (pH-7.5) and homogenized by using Teflon homogenizer. The homogenate was used for the estimation of thiobarbituric acid reactive substances (TBARSs).

##### TBARS assay

The LPO of *C. asiatica* extract was determined according to the method of Kumari et al. (2016). Liver homogenate (0.25 mL) was mixed with 0.1 mL Tris HCL buffer (pH 7.2), 0.05 mL of 0.1 mM ascorbic acid, 0.05 mL 4 mM FeCl_2_ solution and 0.05 mL of the test extracts. All extracts were tested at five different concentrations (4–64 μg/mL). The mixture was incubated at 37°C for 1 h and then 1.5 mL 0.8% (w/v) 2- thiobarbituric acid, 1.5 mL 20% acetic acid, and 0.2 mL 8.1% (w/v) sodium dodecyl sulfate were added to the reaction mixture. The mixture was made up to 4.0 mL with distilled water and heated at 95°C for 60 min. After cooling with tap water, 1.0 mL distilled water and 5.0 mL of a mixture of n-butanol and pyridine (15:1, v/v) were added. The mixture was shaken vigorously. The absorbance was measured at 532 nm in a spectrophotometer (Beckman, UK). Ascorbic acid and trolox were taken as a standard.

### *In vivo* Study

#### Experimental Animals and Ethics Statement

All the experiments were conducted by using laboratory-bred male Wistar albino rats at IASST, Guwahati, Assam, in accordance with the internationally accepted guideline for experimental animals use and the study was approved by the Institute Animal Ethics Committee (IAEC) (1706/GO/c/13/CPCSEA), IASST. All animals were housed under controlled conditions of temperature (24 ± 3°C), relative humidity (60 ± 10%), 12/12 h. light–dark cycle, and water *ad libitum* the animal house of the IASST. Wister rats (male) weighing between 150 and 200 g were utilized for animal experiments.

#### Acute Toxicity Study

Acute toxicity test was conducted in C3H mice (*n* = 6) following the Organization for Economic Co-operation and Development (OECD) protocol. The animals were kept fasting overnight except water *ad libitum* and administered with a single dose of 5 mg/kg body weight (b.w.) with under observation for a period of 14 days. As per the protocol mandate, (i) if mortality was observed in two out of three animals, then the dose administered was assigned as toxic dose, (ii) If mortality took place for single out of three, the dose was repeated to confirm the toxicity, (iii) if there was no mortality at all, the dose specificity had been raised to the maximum of 2,000 mg/kg b.w.

#### Induction of Hyperlipidemic, Experimental Design, and Treatment Schedule

For the development of hyperlipidemic conditions, rats weighing between 150 and 200 g were fed with high-cholesterol-diet (HCD), consisting of cholesterol 2%, whole wheat 62.5 g, yellow corn 37.5 g, barley, vitamin B_12_ one tablet. The cholesterol solution was prepared under the requirement of 25 mg/kg b.w. of rat by dissolving the cholesterol in refined groundnut oil (0.5% w/v) (Khanna et al., 2002). Each individual animal was given 12 g of diet per day. All groups were subjected to intragastric administration every day till 28 days. After allowing a week for acclimatization, the rats were divided into six groups (six rats per group). Group A: normal diet and water (control). Group B: normal diet + cholesterol (25 mg/kg b. w.)/day. Group C: normal diet + cholesterol (25 mg/kg b.w./day) + fenofibrate (65 mg/kg b.w./day). Group D: normal diet + cholesterol (25 mg/kg b.w./day) + CAE_1_ (0.25 g/kg b.w./day). Group E: normal diet+ cholesterol (25 mg/kg b.w./day) + CAE_2_ (0.5 g/kg b.w./day). Group F: normal diet+ cholesterol (25 mg/kg b.w./day) + CAE_3_ (1 g/ kg b.w./day) up to 28 days.

#### Collection of Heart, Liver, and Serum

At the end of the experiment, all feed was removed 14 h before anesthesia. After being anesthetized, the heart, liver, kidney from rat were removed and weighed. The livers were collected and stored at -80°C for further analyses. Blood samples were also collected by an intracardiac puncture followed by separation of serum from the blood samples and collection in heparinized tubes. The collected serum samples were mixed gently by inverting 2–3 times and incubation at 4°C for 2–3 h. Plasma was also separated from blood by centrifugation at 2500 rpm for 30 min, which was aliquoted and stored at 4°C until further use (Feng et al., 2015).

#### Preparation of Tissue Homogenate

Tissue homogenate (rat brain, liver, heart, and kidney) was prepared in a ratio of 1 g of wet tissue to 10 times (w/v) 0.05 M ice-cold phosphate buffer (pH 7.4) and homogenized by using a Teflon homogenizer. 0.2 mL of homogenate was used for estimation of TBARS. The remaining part of the homogenate was divided into two parts, one part of which was mixed with 10% TCA (1:1), centrifuged at 5000 rpm (4°C, for 10 min) and the supernatant was used for GSH estimation. The other part of the homogenate was centrifuged at 15000 rpm at 4°C for 60 min, the supernatants were used for SOD estimation.

#### Biochemical Analysis

##### Blood lipid profile analysis

Total cholesterol, TG, and HDL-C in serum were determined using test kits from Accurex Biomedical Pvt. Limited. Low density lipoprotein cholesterol (LDL-C) and very low density lipoprotein cholesterol (VLDL-C) were calculated using Friedwald’s formula (Friedwald’s et al., 1972).

##### Evaluation of tissue markers of oxidative stress

Thiobarbituric acid reactive substances were measured as a marker of lipid peroxidation for plasma, heart, and liver tissues by using the procedure described by Ohkawa et al. (1979), while glutathione (GSH) by Ellman (1959) and superoxide dismutase (SOD) (Marklund and Marklund, 1974). Nitrate/nitrites (NO) level was assayed by using the method described by Green et al. (1982).

#### Histopathological Evaluation

Rats were fed diets for 4 weeks. Upon termination of the experiment, food was withheld from the rat for 24 h before sacrifice. Rat were then anesthetized, and livers were immediately excised, weighed and stored at 80°C for further use, or fixed in 10% buffered formalin at room temperature for histological analysis. For hepatic histological examination, formalin-fixed liver samples were embedded in paraffin, sectioned and stained with hematoxylin and eosin (H&E), and subjected to light microscope observation.

### Statistical Analysis

The statistical analysis was carried out using the OriginPro 9.0 software packages (OriginLab Corporation, Northampton, MA, USA) and the statistical Pearson’s correlation coefficients by using the OriginPro 6.0 software packages (OriginLab Corporation, Northampton, MA, USA). The results were determined by using one-way ANOVA and *p* < 0.05 were set as significant. IC_50_ was calculated using GraphPad PRISM, version 6.03 for windows (GraphPad software). All the measurements were performed in triplicate (*n* = 3). Mean values ± SD were calculated.

## Results

### Identification of Phenolic Compounds

**FIGURE 1:**
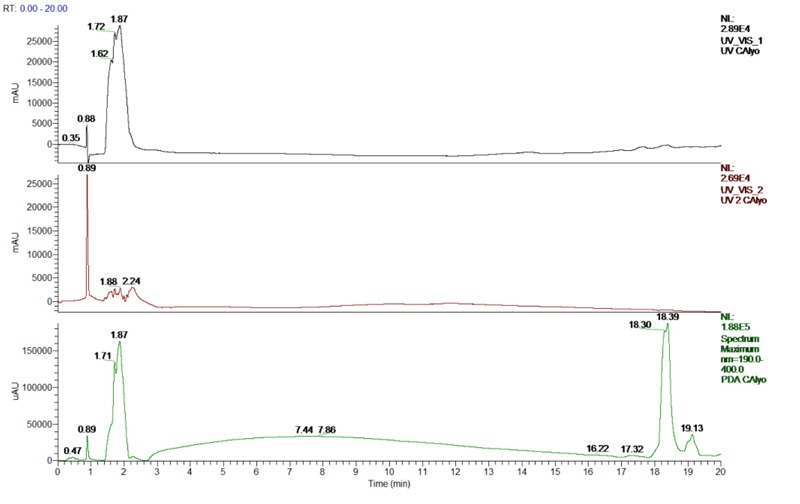
**Ultra-high performance liquid chromatography (UHPLC) chromatogram at 265, 354, 190–400 nm range of *C. asiatica* extract**.

The screening and identification of *C. asiatica* extract was performed by UHPLC–ESI-MS/MS. The chromatographic runs are illustrated in **Figure 1** and Supplementary Figure S1. It reveals the observation of intense peaks at 0–20 min. The peak identification was performed by comparison of the retention time (RT), λ max, and mass spectra of the *C. asiatica* with the standard compounds and the earlier literature reports. Peaks with RTs (min) of 1.62, 1.72, 1.87, 1.88, 2.24 (peaks 1–5) were identified as the following: gulonic acid (RT 1.62, [M-H]- ion at m/z 195.81, λ max 275 nm), ferulic acid (RT 1.72, [M-H]^-^ ion at m/z 193.81, λ max 275 nm), kaempferol (RT 1.87, [M+H]^+^ ion at m/z 287.89, λ max 275 nm), chlorogenic acid (RT 1.88, [M-H]^-^ ion at m/z 353.09, λ max 275 nm), and asiatic acid (RT 2.24, [M-H]^-^ ion at m/z 487.34, λ max 275 nm).

### *In vitro* Antioxidant Activities

#### DPPH Radical Scavenging Activity

**FIGURE 2:**
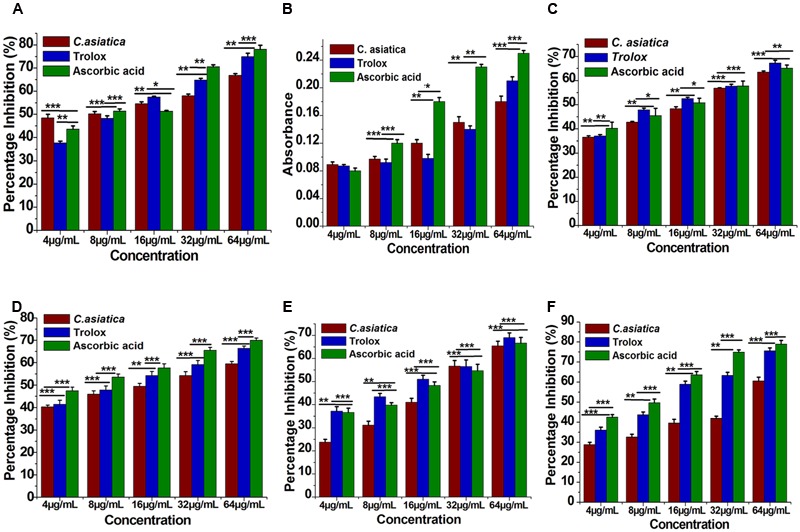
**Antioxidant activity of *C. asiatica* extract at different concentration (4–64 μg/mL). (A)** 2-2- Diphenyl-1-picryl-hydrazyl (DPPH) free radical scavenging activity. **(B)** Total reductive capability. **(C)** ABTS radical radical-scavenging ability. **(D)** H_2_O_2_ radical scavenging activity. **(E)** Nitric oxide (NO) radical-scavenging ability. **(F)** Lipid peroxidation assay. Each point represents the mean ± SD (*n* = 3). ^∗∗∗, ∗∗^, and ^∗^ shows statistical significant differences at *p* < 0.001, *p* < 0.01, and *p* < 0.05.

**FIGURE 3:**
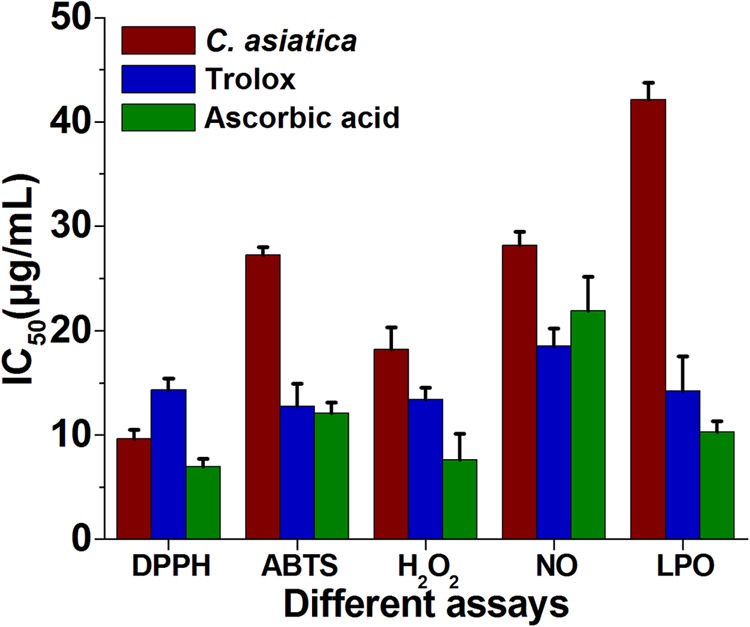
**The IC_50_ (μg/mL) values of *C. asiatica* extracts for free radical scavenging activity by DPPH, ABTS, H_2_O_2_, NO, and LPO radical (Lower IC_50_ value indicates higher antioxidant activity)**.

The reduction capability of DPPH radicals was determined by the decrease in its absorbance at 517 nm induced by extracts having antioxidant potential. It produced hydrazine by converting the unpaired electrons to paired electron due to the hydrogen donating ability of the extract (Ozsoy et al., 2008). As shown in **Figure 2A**, at a concentration of 64 μg/mL, the IC_50_ values of water extracts of *C. asiatica*, trolox, and ascorbic acid were 9.62 ± 0.88, 14.32 ± 1.6, and 6.93 ± 0.76 μg/mL, respectively. In this present study, the IC_50_ value of *C. asiatica* extract was demonstrated significantly higher free radical scavenging activity compared to the standard, trolox while the lowering IC_50_ value indicates a higher free radical scavenging activity (**Figure 3**).

#### Total Reduction Capability

The reducing ability of the extract was determined by the methods (Ahmed et al., 2013; Kumari et al., 2016). The reduction capability of the tested plant extract was increased with the increasing concentration in trolox and ascorbic acid equivalent as shown in **Figure 2B.** From the analysis, the absorbance of *C. asiatica* at 64 μg/mL was 0.18 respectively. Trolox and ascorbic acid were used as positive control and its reducing power at 64 μg/mL were 0.21 and 0.28. The results showed a concentration-dependent significant increase (*p* < 0.05) in the reductive ability of the test samples. These results demonstrated that *C. asiatica* extract had marked the difference in ferric ions (Fe^3+^) reducing ability as compared to the ascorbic acid.

#### ABTS Radical Cation Decolourization Assay

The ABTS^+^ radical scavenging ability is an important method for determining the antioxidant ability. ABTS, a protonated radical, has characteristic absorbance maxima at 734 nm, which decreases with the scavenging capacity (Wang et al., 1998). The results from the ABTS^+^ radical scavenging ability was found to be high in *C. asiatica* (IC_50_ = 27.21 μg/mL) followed by ascorbic acid (IC_50_ = 12.06 ± 1.06 μg/mL) and trolox (12.76 ± 2.1 μg/mL) (**Figure 3**). *C. asiatica*, ascorbic acid and trolox exhibited the dose-dependent effective antioxidant activity (**Figure 2C**).

#### Hydrogen Peroxide (H_2_O_2_) Radical Scavenging Activity

Hydroxyl radicals are extremely reactive free radicals formed in the biological system and there is no any specific enzymes to defend against them in human (Liu et al., 2005). The scavenging ability of *C. asiatica*, ascorbic acid and trolox are shown in **Figure 2D**. H_2_O_2_ radical scavenging ability of extracts of *C. asiatica*, ascorbic acid and trolox at the concentration of 64 μg/mL were 59.93 ± 1.07, 69.96 ± 1.07, and 66.43 ± 1.4%, respectively. The IC_50_ values of *C. asiatica*, ascorbic acid and trolox found to be 18.23 ± 2.1, 7.6 ± 2.51, and 13.43 ± 1.4 μg/mL, respectively. The IC_50_ value indicates that the plant extract is a better hydroxyl radical scavenger, which quite comparable with the standard ascorbic acid and trolox (**Figure 3**). In our study, the hydroxyl radical scavenging activity was increased by the extract with increasing concentration.

#### Nitric Oxide (NO) Radical Scavenging Activity

From the analysis, the water extract of *C. asiatica* showed the highest inhibitory effect with the IC_50_ value of 28.15 ± 2.01 μg/mL at the concentration of 64 μg/mL. In contrast, trolox and ascorbic acid showed the inhibitory effect with the IC_50_ value 18.53 ± 1.7, and 21.93 ± 3.2 μg/mL, respectively (**Figure 3**). Therefore, NO radical scavenging activity of *C. asiatica* was quite comparable to trolox and ascorbic acid at the concentration of 64 μg/mL (**Figure 2E**). Earlier reports demonstrate that the phenolic compounds play a vital role in NO suppression, which might be the reason behind differential inhibitory effect observed in this study (Kumari et al., 2016).

#### Inhibition of Lipid Peroxidation (LPO)

In this assay, LPO was significantly inhibited by plant extracts in a dose-dependent manner (**Figure 2F**). The inhibition at the concentration of 64 μg/mL is followed in the order, *C. asiatica*< trolox < Ascorbic acid. *C. asiatica* showed the highest inhibition of LPO with 60.53 ± 2.21% (*p* < 0.05), at the concentration of 64 μg/mL whereas trolox showed 75.66 ± 1.45% of inhibitory effects. Additionally, ascorbic acid was also utilized as a positive control significantly inhibited LPO by 78.93 ± 1.86%. In addition, the IC_50_ values of *C. asiatica*, trolox, and ascorbic acid were recorded to be 42.13 ± 2.32, 14.21 ± 1.01, and 10.31 ± 1.03 μg/mL, respectively (**Figure 3**). It has been reported that phenolic compounds have shown more potential to prevent LPO and the associated diseases (Kumari et al., 2016). The results had clearly corroborated the efficacy of *C. asiatica* as a promising source of inhibiting LPO.

### Acute Toxicity and Effect of CAE on Body Weight

The results of the acute oral administration of CAE in various doses up to 2000 mg/kg indicated no mortality up to 15 days after treatment. According to the acute toxicity studies of the ethanolic extract of CAE at a dose ranging from 2 to 5 g per kg body weight for 14 days did not manifest any significant and noticeable signs of toxicity in rats. In this study, after 28 days, the body weight of groups including normal or HCF feeding was significantly affected. The changes in body weight are shown in **Table 2**. The body weight was significantly increased in case of HCF fed groups than rats subjected to normal conditions (NCs) (two-way ANOVA, *p* < 0.05). However, the body weight of hyperlipidemic rat treated with the three different dose levels of CAE (CAE_1_ = 0.25 g/kg b.w./day, CAE_2_ = 0.5 g/kg b.w./day, CAE_3_ = 1 g/kg b.w./day) demonstrated a remarkable decrease compared to the HCF group (one-way ANOVA, *p* < 0.05). This result was in agreement with previous work by who observed a marked decrease in body weight in CAE treated rats.

**Table 1:** Pearson’s correlation coefficients between the five assays.

	DPPH	ABTS^+^	H_2_0_2_	NO	LPO
ABTS^+^	0.969ˆ*	–	0.996ˆ***	0.997ˆ***	0.937ˆ*
H_2_0_2_	0.965ˆ**	0.996ˆ***	–	0.988ˆ***	0.942ˆ**
DPPH	–	0.969ˆ*	0.965ˆ*	0.965ˆ*	0.991ˆ***
NO	0.965ˆ*	0.997ˆ***	0.988ˆ***	–	0.926ˆ*
LPO	0.991ˆ**	0.937ˆ*	0.942ˆ*	0.926ˆ*	–

*^∗^Correlation is significant at the 0.05 level.*

*^∗∗^Correlation is significant at the 0.01 level.*

*^∗∗∗^Correlation is significant at the 0.001 level.*

**Table 2:** Effect of various extracts of CAE (*Centella asiatica* extract) on body weight (b.w.) gram (g) in HCF- induced rats.

Different group	Initial body weight (1st day)	Final body weight (28th day)
NC	173.5 ± 2.21	180.2 ± 3.53
HCF	188.2 ± 2.85	208.1 ± 3.44
HCF+FF	177.2 ± 4.23	182.3 ± 3.31
HCF+CAE_1_	175.3 ± 3.43	189.2 ± 2.73
HCF+CAE_2_	178.22 ± 2.11	187.3 ± 3.51
HCF+CAE_3_	175.22 ± 3.41	184.41 ± 2.97

*CAE, *Centella asiatica* extract; NC, normal control; HCF, high cholesterol-fed; HCF+FF, high cholesterol-fed + fenofibrate; HCF+CAE_*1*_, high cholesterol-fed + *Centella asiatica* extract_*1*_ (0.25 g/kg b.w./day); HCF+CAE_*2*_, high cholesterol-fed + *Centella asiatica* extract_*2*_ (0.5 g/kg b.w./day); HCF+CAE_*3*_, high cholesterol-fed + *Centella asiatica* extract_*3*_ (1 g/kg b.w./day).*

### Biochemical Parameters

As shown in **Table 3**, serum lipids level (TC, TG, VLDL-C, and LDL-C) were increased significantly in HCF group in comparison to that of NC group (*p*< 0.05). Treatment with three different dose levels of CAE(CAE_1_ = 0.25 g/kg b.w./day, CAE_2_ = 0.5 g/kg b.w./day, CAE_3_ = 1 g/kg b.w./day)in the HCF diet significantly lowered (*p*< 0.05) plasma TC by 35.7, 39.81, and 51.72%, respectively and TG by 24.83, 32.15, and 38.83%, respectively compared to the HCF groups. As far as the serum, treatment with CAE_1_, CAE_2_, and CAE_3_ elevated the HDL-C level and decline in the LDL-C contents. It was noted that CAE exhibited comparable potencies with those of fenofibrate on lipid parameters. Several studies have shown that TC, TC, TG, VLDL-C, and LDL-C level as biomarkers for hyperlipidemia (Stein and Stein, 1999; Devi and Sharma, 2004). In this present work, CAE also improved the lipid profile, which was quite comparable with other studies (Stein and Stein, 1999; Devi and Sharma, 2004).

**Table 3:** Effect of various extracts of CAE (*C. asiatica* extract) on rats’ serum lipid profile.

Parameters (mg/dL)	NC	HCF	HCF+FF	HCF+CAE_1_	HCF+CAE_2_	HCF+CAE_3_
TC	75.04 ± 3.23	162.52 ± 5.5^++^	126.55 ± 6.6^∗^	104.4 ± 3.21^∗^	97.69 ± 3.42^∗^	78.43 ± 2.46^∗∗^
TG	64.47 ± 3.31	117.7 ± 4.11^+^	97.34 ± 4.47	88.47 ± 3.2	79.85 ± 2.03^∗^	72.01 ± 2.46^∗∗^
HDL	64.69 ± 3.42	36.55 ± 4.91^+^	42.73 ± 2.12	57.30 ± 3.2	59.03 ± 4.21^∗^	60.71 ± 5.11^∗^
LDL	53.2 ± 2.3^++^	138.92 ± 4.2^++^	83.54 ± 7.21	58.13 ± 5.6^∗^	56.41 ± 3.29^∗^	54.73 ± 3.45^∗∗^
VLDL	16.83 ± 2.72	26.37 ± 3.96^+^	21.44 ± 3.94	19.23 ± 2.1	18.78 ± 1.03	15.41 ± 2.33^∗^

*CAE, *Centella asiatica* extract; NC, normal control; HCF, high cholesterol-fed; HCF+FF, high cholesterol-fed + fenofibrate; HCF+CAE_*1*_, high cholesterol-fed + *Centella asiatica* extract_*1*_ (0.25 g/kg b.w./day); HCF+CAE_*2*_, high cholesterol-fed + *Centella asiatica* extract_*2*_ (0.5 g/kg b.w./day); HCF+CAE_*3*_, high cholesterol-fed + *Centella asiatica* extract_*3*_ (1 g/kg b.w./day).*

*^∗^*p* < 0.05 vs. HCF group, ^∗∗^*p* < 0.01 vs. HCF group, ^+^*p* < 0.05 vs. NC group, ^++^*p* < 0.01 vs. NC group. *n* = 6, mean ± SEM.*

### Oxidative Stress Markers

In hyperlipidemic condition, enzymatic as well as non-enzymatic antioxidative defense system such as SOD and reduced GSH are altered leading to reactive oxygen species (ROS) mediated damage (Devi and Sharma, 2004). As shown in **Table 4**, the OS markers (SOD, GSH, TBARS, and NO) levels increased significantly (*p*< 0.05) in serum, heart, and liver of HCF rats in comparison to that of NC group.

**Table 4:** Changes in oxidative stress markers (SOD, CAT, GSH, TBARS, and NO) in heart, liver, and serum.

Parameters		NC	HCF	HCF+FF	HCF+CAE_1_	HCF+CAE_2_	HCF+CAE1
Heart	SOD^a^	69.45 ± 3.4	38.16 ± 1.15^+^	65.39 ± 2.26	61.16 ± 0.01	64.99 ± 1.5	67.99 ± 1.5^∗^
	GSH^b^	112 ± 5.6	60 ± 3.2^++^	80 ± 4	81.82 ± 2.7^∗^	87 ± 6.1^∗∗^	89 ± 6.21^∗∗^
	TBARS^c^	35.2 ± 1.6	52.32 ± 7.2^+^	41.1 ± 2.3	39.2 ± 3.2	38.61 ± 2.5	38.18 ± 2.1
	NO^d^	15.18 ± 1.34	27.14 ± 1.32	17.74 ± 1.31	21.32 ± 2.21	17.21 ± 1.0	13.21 ± 1.0
Liver	SOD^a^	86.35 ± 1.21	55.14 ± 1.11^+^	79.26 ± 1.03	70.31 ± 1.12	79.96 ± 2.2	81.16 ± 1.2^∗∗^
	GSH^b^	350 ± 10	258 ± 6.5^+^	344 ± 4.5	318.17 ± 8.5	336 ± 6.7	356 ± 6.6^∗^
	TBARS^c^	76.4 ± 5.3	187.2 ± 7.1^++^	147.57 ± 8.1	116.2 ± 6.7	97.66 ± 7.230.6	82.4.7 ± 6.7^∗∗^
	NO^d^	21.47 ± 2.31	47 ± 3.26	23.64 ± 2.43	33.34 ± 2.7	5 ± 2.5	24.65 ± 0.5
Serum	SOD^a^	32.13 ± 1.13	19.49 ± 2^++^	22.43 ± 1.7	26.56 ± 5.23	28.32 ± 4.2	32.25 ± 2.1^∗∗^
	GSH^e^	132 ± 5.3	201 ± 7.3	104 ± 4	114.89 ± 2.7	124.5 ± 5	130.2 ± 7.5
	TBARS^f^	29.2 ± 1.4	46.92 ± 3.6	37.37 ± 4.2	33.1 ± 2.27	27.62 ± 1.9	28.81 ± 2.1
	NO^g^	26.63 ± 1.51	56.56 ± 1.21	23.82 ± 1.51	39.51 ± 2.12	33.19 ± 1.1	25.23 ± 4.3

*CAE, *Centella asiatica* extract; NC, normal control; HCF, high cholesterol-fed; HCF+FF, high cholesterol-fed + fenofibrate; HCF+CAE_*1*_, high cholesterol-fed + *Centella asiatica* extract_*1*_ (0.25 g/kg b.w./day); HCF+CAE_*2*_, high cholesterol-fed + *Centella asiatica* extract_*2*_ (0.5 g/kg b.w./day); HCF+CAE_*3*_, high cholesterol-fed + *Centella asiatica* extract_*3*_ (1 g/kg b.w./day).*

*^a^% Inhibition, ^b^μg/mg, ^c^μM/mg, ^d^μM/mg, ^e^μg/mL, ^f^μM/ mL, ^g^μM/ mL. ^∗^*p* < 0.05 vs. HCF group, ^∗∗^*p* < 0.01 vs. HCF group, ^+^*p* < 0.05 vs. NC group, ^++^*p* < 0.01 vs. NC group. *n* = 6, mean ± SEM.*

#### Heart

The level of SOD and GSH decreases significantly (*p*< 0.05) in stress induced HCF group heart tissue by 45 and 26.2%, respectively when compared with those of NC groups. CAE treatment groups prevented the alterations in SOD and GSH levels in HCF group heart tissue homogenates, however, CAE_1_, CAE_2_, and CAE_3_ treatment group significantly increase (*p*< 0.01) the serum SOD level by 60.2, 70.3, and 78.1%, respectively. In addition, CAE_1_, CAE_2_, and CAE_3_ and significantly increases (*p*< 0.01) the GSH level by 35.2, 45.5, and 48.3%, respectively. However, there were no significant differences in the other measured parameters.

#### Serum

In serum of HCF rat, the SOD level decreases significantly (*p*< 0.01) by 39.34% compared with those of NC group. CAE_3_ treatment group significantly increases (*p*< 0.01) the serum SOD level by 65.4%. However, CAE_1_ and CAE_2_ treatment did not incorporate any significant changes in serum SOD parameters. However, there were no significant differences were detected in the other measured parameters.

#### Liver

The level of SOD and GSH were decreased significantly (*p*< 0.05) and TBARS increase significantly (*p*< 0.05) in liver homogenates of HCF group when compared with those of the NC group. CAE_1_, CAE_2_, and CAE_3_ treatment groups prevented the alterations in the level of SOD and GSH in HCF group. CAE_1_, CAE_2_, and CAE_3_ increases the SOD (30.21, 45.1, and 47.12%) and GSH (23.3, 30.21, and 37.23) level. TBARS level decreases significantly (*p*< 0.05) by 55.59%. No changes were observed in the level of NO in CAE treated groups.

### Histopathological Observations in Liver Tissue Sections

**FIGURE 4:**
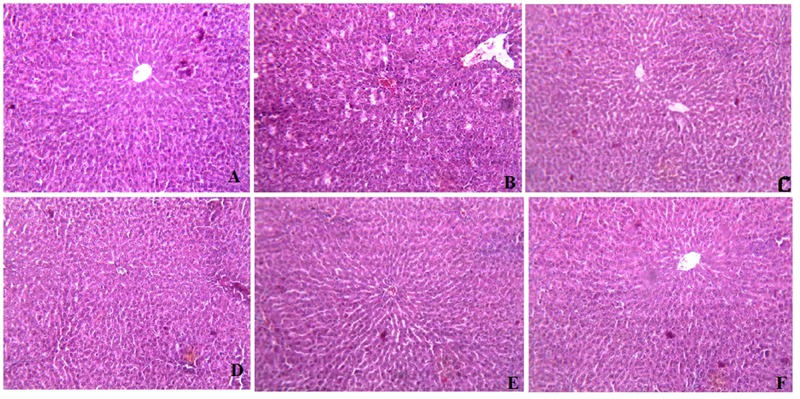
**Histopathological effects of *C. asiatica* extracts (CAE) in liver of High cholesterol-fed (HCF) rat (A) Represents Hematoxylin Eosine staining of normal liver with glomerulus. (B)** High cholesterol-fed (HCF) liver with large area of necrosis, congested central vein (CV) and lipid droplets (LD) accumulation. **(C)** Fenofibrate treated liver showing restoration of hepatic structure. **(D)** CAE_1_ (0.25 g/kg) treated liver; **(E)** CAE_2_ (0.5 g/kg) treated liver; **(F)** CAE_3_ (0.1 g/kg) treated liver; depicting improvement in the hepatic structure and sufficient reduction in the appearance of LD.

To further investigate hepatocyte morphological changes during the function of CAE, liver tissue sections using histopathological microscopy were examined. As shown in **Figure 4**, after 28 days of treatment with CAE, the liver cell structure of HCF rats in the high dose CAE treated group was integrated. The sizes of lipid droplets in the CAE group were remarkably smaller than those of HCF group, suggesting that CAE could reduce the accumulation of lipid droplets. In the present study, these data directly confirm that CAE treatment group can keep hepatocytes normal by preventing or reducing excess lipid formation in HCF rat.

## Discussion

The findings of this study demonstrated the antioxidant and anti-hyperlipidemic ability of extracts of *C. asiatica* by several *in vitro* and *in vivo* methods. The experimental results showed that extract of *C. asiatica* showed strong DPPH scavenging activity. Earlier reports demonstrated the IC_50_ value of *C. asiatica* in DPPH radical scavenging assay was in the range of 31.25 μg/mL (Pittella et al., 2009; Ahmed et al., 2013). However, in this study, the IC_50_ value of *C. asiatica* in DPPH radical scavenging assay was far lower (∼8 μg/mL) in comparison with the previous report (Pittella et al., 2009; Ahmed et al., 2013). Several previous reports demonstrated that the presences of the antioxidants in the extract were determined by assessing the ability of extract to reduce the ferric cyanide complex to the ferrous form (Ahmed et al., 2013; Kumari et al., 2016). The reducing power of a compound may serve as a significant indicator of its potential antioxidant activity (Pal et al., 2011). Samples with higher reducing power have better abilities to donate the electron and the free radical form stable substances by accepting the donated electrons, resulting in the termination of radical chain reaction (Deori et al., 2014). In our previous report, the polyphenolic compounds of *C. asiatica* showed significantly reduced the ferric cyanide complex to the ferrous form (reducing capabilities) at higher concentrations (Kumari et al., 2016). In the present study, the reducing ability of the extracts of *C. asiatica* was quite comparable (**Figure 2B**) with previous studies (Ahmed et al., 2013; Kumari et al., 2016). The previous report confirmed that the hydroxyl radicals are extremely reactive free radicals formed in the biological system and there are no any specific enzymes to defend against them in human (Liu et al., 2005). The presence of hydroxyl radical in the body may lead to the oxidative DNA damage. Therefore, it is very important to find the solution using natural products with good scavenging activity against this ROS. Earlier report also demonstrates that the scavenging of hydroxyl radical is an important antioxidant activity because of very high reactivity of the OH radical (Wang et al., 2008). In our study, each extract showed an increase in hydroxyl radical scavenging activity with increasing concentration of sample extracts. Overproduction of NO causes cancer, inflammation, neurodegenerative, chronic inflammatory diseases, ischemia-reperfusion and other pathological conditions (Moncada et al., 1991; Bajpai et al., 2014). Earlier reports demonstrate that phenolic compounds play a vital role in NO suppression, which might be the reason behind the differential inhibitory effect, observed in this study (Kumari et al., 2016). In the present study, the extracts scavenging the NO which results in the reduction of chromophore formation, and decreases the absorbance with the increase in extract concentrations. We further confirm that the ABTS^+^ radical scavenging activity of the extract of *C. asiatica* showed a good radical scavenging capacity with low IC_50_ value. LPO involves the generation of free radical and hydroxyl radical are the major active oxygen species causing LPO and enormous biological damage (Chidambaram et al., 2013). It is believed that LPO is one of the major causes of cardiovascular disease and cancer, generating malondialdehyde (MDA) like product during through a set of chemical reaction (Atmani et al., 2009). In the present study, LPO was significantly inhibited by all the extracts a dose-dependent manner. The inhibition at the concentration of 64 μg/mL followed order; *C. asiatica*< trolox < Ascorbic acid. The results had clearly corroborated the efficacy of *C. asiatica* as a promising source of inhibiting LPO. Earlier study demonstrated that the strong correlation between DPPH and ABTS methods (Floegel et al., 2011). In the present study, **Table 1** shows the correlation between the five assays. DPPH was significantly and positively correlated with ABTS^+^, H_2_O_2_, NO, and LPO. In the cases of H_2_O_2_, indicates strongly correlated with ABTS^+^ and NO. Additionally, ABTS^+^ and NO were significantly correlated with H_2_O_2_, but not DPPH and LPO.

Elevation of LDL-C and TC and reduction of high density lipoprotein level is associated with progression of atherosclerotic lesions (Harrison et al., 2003; Minhajuddin et al., 2005; Jain et al., 2007). In various studies, it has been reported that HCF rat significantly increased (*p* < 0.05) the levels of TC, HDL, LDL, VLDL, TGs, and also significantly decreased the level of HDL in the serum (Devi and Sharma, 2004). In this present work, CAE also improved the lipid profile, which was quite comparable with other studies (Stein and Stein, 1999; Devi and Sharma, 2004). In hyperlipidemic condition, enzymatic as well as non-enzymatic antioxidative defense system such as SOD and reduced GSH is altered leading to ROS mediated damage (Devi and Sharma, 2004). As shown in **Table 4**, the OS markers (SOD, GSH, TBARS, and NO) levels increased significantly (*p*< 0.05) in serum, heart, and liver of HCF rats in comparison to that of NC group. Earlier report also demonstrates that hypercholesterolemia diminishes the antioxidant defense system and decreases the activities of SOD and catalase in rats (Fki et al., 2005). Enzymes such as SOD and catalase which contribute to the antioxidant defense mechanism (Lee et al., 2002). However, in the present investigation, treatment with CAE increases the efficiency of SOD and GSH. Taken together, our result suggested that the administration of CAE could improve the efficiency of SOD and GSH. Furthermore, the previous report demonstrates that there is an increase of TBARS in the animal fed with a high cholesterol diet (Jiang et al., 2015). In addition, the decreased in the activity of these antioxidants can lead to an excess availability of superoxide anion and H_2_O_2_ in biological systems, which in turn generate hydroxyl radicals resulting in initiation and propagation of LPO (Kumuhekar and Katyane, 1992). In the present study, the similar increasing trend in TBARS level in HCF rat was observed. Herein, there was the preventive effect on the lipid peroxidation level by treatment with CAE extract. Earlier report also demonstrates that lipid drops are usually accumulated in hepatic tissue, called hepatic steatosis, under the progress of atherosclerosis, especially hyperlipidemia stage. However, enzymes such as SOD and catalase which contribute to the antioxidant defense mechanism and reducing excess lipid drops (Lee et al., 2002; Devi and Sharma, 2004). In the present study, these data directly confirm that CAE treatment group can keep hepatocytes normal by preventing or reducing excess lipid formation in HCF rat.

## Conclusion

In this study, we have evaluated the antioxidant and anti-hyperlipidemic ability of extracts of *C. asiatica*. Herein, *C. asiatica* showed promising antioxidant and -antihyperlipidemic activities and also exhibited the highest phytochemical contents. The results of the present study indicate the presence of strong phenolic antioxidants components mainly gulonic acid, ferulic acid, kaempferol, chlorogenic acid, and asiatic acid in *C. asiatica* extract as evidenced from UHPLC-MS/MS. Additionally, CAE is capable of exhibiting significant anti-hyperlipidemic activities in HCF induced rat by enhancing parameters like antioxidant enzyme, body weight, and decrease serum lipid levels as well as regeneration of hepatic structures. Moreover, the CAE exerted to improve the hyperlipidemia induced hepatic structures by reducing OS, and restoring the antioxidant capacities. Taken together, this study strongly suggests that the CAE treatment might be an efficient way for treatment hyperlipidemia. This study has provided more evidence for the use of *C. asiatica* as a promising traditional medicine in the therapy of hyperlipidemia. Thus, these plant leaf extracts may be utilized as natural agents in food and pharmaceutical industries. Further studies are needed to isolate and identify the bioactive compounds present in the plant extracts and for the elucidation of their molecular mechanisms.

## Author Contributions

SK conceived and designed the experiment. SK, MD, RE performed the experiment. SK analyzed the data. SK and RE wrote the manuscript. JK and RD have done a critical revision of the manuscript for important intellectual content. RD has been the corresponding author and MD and RE shared as second authors. All authors have contributed to the final version and approved the final manuscript.

## Conflict of Interest Statement

The authors declare that the research was conducted in the absence of any commercial or financial relationships that could be construed as a potential conflict of interest.
