# Supplementary material for: In vitro and In vivo Antioxidant, Anti-hyperlipidemic Properties and Chemical Characterization of Centella asiatica (L.) Extract
Source: Front Pharmacol. 2016 Oct 28;7:400. doi: 10.3389/fphar.2016.00400 (PMC5083837; doi:10.3389/fphar.2016.00400)
Supplement: Supplementary file 1 [file Data_Sheet_1.DOCX]

**SUPPORTING INFORMATION**

***In vitro* and *in vivo* antioxidant, anti-hyperlipidemic properties and chemical characterization of *Centella asiatica* (L.) extract**

Sima Kumari^1^, Meetali Deori^1^, R. Elancheran^2^, Jibon Kotoky^2^, Rajlakshmi Devi^1*^

^1^ Biochemistry Laboratory, Life Sciences Division, Institute of Advanced Study in Science and Technology, Guwahati-781035, Assam, India

^2^ Drug Discovery Laboratory, Life Sciences Division, Institute of Advanced Study in Science and Technology, Guwahati-781035, Assam, India

*Corresponding author: Tel: +(91)-361-2912073

E-mail address: [rajiasst@gmail.com](mailto:rajiasst@gmail.com)

Figure

Fig. Total Ion Chromatogram (TIC) at positive and negative as well as fragmentation with higher-energy collisional dissociation at 40eV in both modes of *C. asiatica* extract

**Methods:**

**Phytochemical content**

**Estimation of total phenolic content (TPC)**

Total phenolic content was determined by Folin-Ciocalteu method with slight modifications (Hagerman, 2002). Briefly, 0.5 mL extracts were mixed with Folin-Ciocalteu reagent (2.5 mL, 10 times diluted) and incubated for 2 mins at room temperature followed by addition of sodium carbonate solution (2 mL, 7.5% w/v). The mixture was then allowed to stand for 30 mins at room temperature then the absorbance was measured at 765 nm. The TPC was calculated as a catechin equivalent from the calibration curve of catechin standard solution and expressed as mg catechin/g dry weight of the sample.

**Estimation of total flavonoids content (TFC)**

The estimation of total flavonoids was carried out according to the aluminum chloride colorimetric assay (Yanpinget al., 2004). An aliquot (1mL) of water extracts or standard solution of quercetin was added to 10mL the volumetric flask containing 4mL of distilled water. To the above mixture, 0.3mL of 5% NaNO_2_ was added. After 5mins, 0.3 mL of 10% AlCl_3_ was added. At 6^th^min, 2mL of 1M NaOH was added and the total volume was made up to 10mL with distilled water. The solution was mixed well and the absorbance was measured against prepared reagent and blank at 510nm. The results were expressed as quercetin equivalent,i.e., expressed as mg quercetin/g dry weight of the sample.

**Estimation of total tannin content (TTC)**

TTC were determined by the Folin-Ciocalteu procedure as described above, after removal of tannins by their adsorption to polyvinyl polypyrrolidone(PVPP) (Liao and Shi, 2005). In brief, 20 mL of water extracts were homogenized with 200 mg of PVPP and the mixture was stirred for 1 hour.After filtration, the supernatant contains only the non-tannin phenolics determined by the Folin-Ciocalteu procedure as described earlier. The calculated values were subtracted from total polyphenols content and the amount of total tannin expressed as mg catechin/g dry weight of the sample.

**Results:**

**Determination of phytochemical content**

Polyphenol is the major secondary metabolites with the high level of antioxidant activity as reported in previous studies (Tsao et al., 2005). Phenolic compounds are biologically active substances, which are considered to be natural antioxidant (Siger et al., 2012). Therefore, it is quite important to determine the total phenolic content in the tested extracts. The total phenolic contents (TPC), total flavonoid content (TFC) and total tannin contents (TTC) of water extracts of *C. asiatica* was calculated using the Folin–Ciocalteu method. The TPC of *C. asiatica* was found to be 16.06 ± 0.83 mg catechin/g of dry weight. TFC and TTC were estimated by using aluminum chloride colorimetric assay and it was observed that *C. asiatica* had significantly higher TFC and TTC(6.16 ± 0. 42 mg quercetin /g dry weight and 2.92 ± 0.11 mg catechin/g of dry weight). Earlier reports demonstrate that with high concentration of phenols in the plant extracts may contribute directly to their antioxidant action (Tosun et al., 2009; Canadanovic-Brunet et al., 2008). Several other studies also showed that polyphenols and other flavonoid possess both hypolipidemic and antihyperlipidemic activity, which arereported for increase of lipoprotein lipase activity. The increased lipoprotein lipase activity further helps to remove free fatty acids from circulation, causing decrease in cholesterol level (Sidhu & Oakenful, 1990). Earlier report demonstrated the total phenolic contents of *A. viridis* were in the range of (2.81-3.61 GAE, g/100 g) andin *C. asiatica*(2.86 g/100g) (Ahmed et al., 2012; Pittella et al., 2009). However, in this study the TPC value of*C. asiatica* and *A. viridis* was higher in comparison with the previous report (Ahmed et al., 2012; Pittella et al., 2009). The results had clearly corroborated the efficacy of *C. asiatica* as a promising and significant source of phytochemical content.
